# Ligands can differentially and temporally modulate GPCR interaction with 14-3-3 isoforms

**DOI:** 10.1016/j.crphar.2022.100123

**Published:** 2022-08-07

**Authors:** Haifeng Eishingdrelo, Xiaofa Qin, Luwa Yuan, Sathapana Kongsamut, Lei Yu

**Affiliations:** a BioInvenu Corp., 50 Williams Parkway, Unit A2, East Hanover, NJ, 07936, USA; b Department of Genetics, and Center of Alcohol & Substance Use Studies, Rutgers University, 145 Bevier Road, LSB 126, Piscataway, NJ, 08854, USA

**Keywords:** G-protein coupled receptor (GPCR), 14-3-3 proteins, Signal transduction, Protein-protein interaction, Receptor trafficking

## Abstract

GPCR signaling and function depend on their associated proteins and subcellular locations. Besides G-proteins and β-arrestins, 14-3-3 proteins participate in GPCR trafficking and signaling, and they connect a large number of diverse proteins to form signaling networks. Multiple 14-3-3 isoforms exist, and a GPCR can differentially interact with different 14-3-3 isoforms in response to agonist treatment. We found that some agonist-induced GPCR/14-3-3 signal intensities can rapidly decrease. We confirmed that this phenomenon of rapidly decreasing agonist-induced GPCR/14-3-3 signal intensity could also be paralleled with GPCR/β-arrestin-2 signals, indicating diminished levels of GPCR/signal adaptor complexes during endocytosis. The temporal signals could implicate either GPCR/14-3-3 complex dissociation or the complex undergoing a degradation process. Furthermore, we found that certain GPCR ligands can regulate GPCR/14-3-3 signals temporally, suggesting a new approach for GPCR drug development by modulating GPCR/14-3-3 signals temporally.

## Highlights

• Some GPCRs can engage or dissociate with different 14-3-3 isoforms in response to agonist treatment.
• Some GPCRs and 14-3-3 isoform interaction signals can be rapidly diminished in response to agonist treatment, the temporal signal strength changes can be paralleled with the same GPCR and β-arrestin-2 interaction signals.
• Adrenergic receptor alpha 2A (ADRA2A) drugs with different therapeutic indications can temporally regulate ADRA2A/14-3-3γ and ADRA2A/β-arrestin-2 interaction complex signals.

## Graphical abstract

**Figure:**
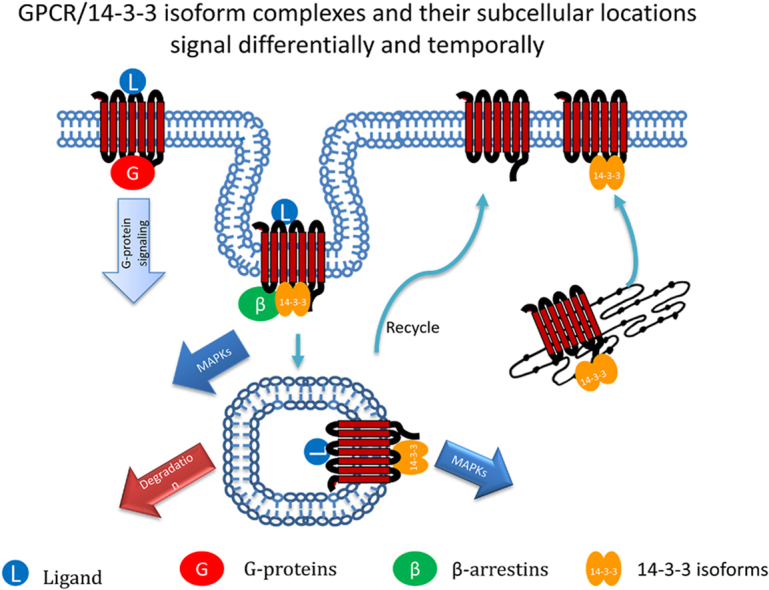

## 1 Introduction

Multiple signaling transduction pathways are employed upon GPCR activation. Receptors can recruit and interact with different cellular effectors and signal adaptors, depending on distinct receptor conformations stabilized by binding with agonists, antagonists, inverse agonists, and biased ligands (Shukla et al., 2008). These associated proteins have profound effects on the overall receptor structure, function, and subcellular localization. Receptor signaling and function depend on the associated proteins and subcellular locations of receptor/associated protein complexes.

GPCRs transduce signals through G-protein subunits. GPCRs can also transduce signals through G-protein-independent pathways. Receptor activation can lead to receptor phosphorylation by G protein-coupled receptor kinases. Phosphorylated receptors can then recruit β-arrestins and produce G protein-independent, β-arrestin-dependent signaling events (Jean-Charles et al., 2017). In contrast to the G-protein signaling pathways which are transient signals, β-arrestins interact with many MAPKs which phosphorylate various nuclear and cytoplasmic proteins to elicit cellular responses (Song et al., 2009; Eishingdrelo et al., 2015).

Besides G-proteins and β-arrestins, an array of signal adaptors or cellular effectors interact with GPCRs. These signal adaptor proteins influence receptor activity, trafficking, subcellular distribution, spatiotemporal signaling, cross-talk with other signaling pathways, and offer the possibility of fine-tuning GPCR signaling at multiple levels (Milligan and White, 2001). One group of cellular proteins associated with GPCRs is 14-3-3 signal adaptor proteins (Li et al., 2016). Similar to the ubiquitously expressed β-arrestins, 14-3-3 proteins have no intrinsic enzymatic activity, but bring two or more proteins together to form signal transduction complexes (Fu et al., 2000; Ferl et al., 2002). The 14-3-3 protein family consists of seven isoforms. They are ∼30-kDa acidic proteins, ubiquitously and abundantly expressed in cells, and are present in the cytoplasm, intracellular organelles, and associated with the plasma membrane (Morrison, 2009). 14-3-3 proteins form homo or heterodimers and function as scaffold proteins to change client protein conformation, facilitate or inhibit client protein interactions, mask or protect client protein phosphorylation, and transport client proteins among different compartments (Fu et al., 2000). A large number of proteins are known to interact with 14-3-3 proteins, including kinases, phosphatases, scaffold proteins, transcription factors, cytoskeletal proteins, and membrane proteins including GPCRs, receptor tyrosine kinases, and ion channels (Foote and Zhou, 2012; Steinacker et al., 2011). These highly complex and intertwined 14-3-3 networks integrate multiple signaling pathways as signal transduction hubs and profoundly regulate cell physiology (Fu et al., 2000; Ferl et al., 2002). Multiple 14-3-3 isoforms may represent one more level of regulation in 14-3-3 signaling, and our knowledge regarding isoform-specific functions is very limited.

Previously, we studied GPCR/14-3-3ε interactions pharmacologically using the LinkLight™ assay technology (Eishingdrelo et al., 2011). We found that GPCR/14-3-3 interaction is phosphorylation-dependent, can be β-arrestin-independent, and can be regulated by GPCR ligands (Li et al., 2016; Yuan et al., 2019). Activation of GPCRs can lead to kinase/14-3-3 interactions, and over 90% GPCRs contain at least one putative 14-3-3 binding site (Yuan et al., 2019; Madeira et al., 2015). Some GPCRs can associate with 14-3-3 proteins at the cell membrane. Agonist binding promotes 14-3-3 protein dissociation and recruits β-arrestins. Some GPCRs that have no 14-3-3 protein bound at the cell membrane can recruit 14-3-3 proteins during or after endocytosis in response to agonist treatment. 14-3-3 scaffold proteins play important roles in directing GPCR recycling and trafficking.

In the present study, we showed, depending on GPCRs, that agonists can promote the recruitment of different 14-3-3 isoforms or recruit one 14-3-3 isoform but dissociate another 14-3-3 isoform. We found in response to agonists, dopamine receptor 2 (DRD2) and serotonin receptor 2a (5HT2A) can recruit both 14-3-3ε and 14-3-3γ isoforms. We also found that the delta-opioid receptor (DOR), in response to agonist treatment, increased DOR/14-3-3γ signals but decreased DOR/14-3-3ε signals. Agonist-induced DOR/14-3-3γ signal intensity quickly diminished while agonist-diminished DOR/14-3-3ε signals took a much longer time to appear. The phenomenon of quickly decreasing agonist-induced DOR/14-3-3γ signal intensity was also observed in the agonist-induced DOR/β-arrestin-2 assay, and in the same timeframe. In contrast, we did not observe agonist-induced MOR/14-3-3γ signals, and MOR/β-arrestin-2 signal intensity gradually increased over time. In addition, we looked at whether different agonists can alter GPCR/14-3-3 signal intensity temporally by using human alpha-adrenergic receptor 2A (ADRA2A) as an example. We found that clonidine, lofexidine, and adrenaline modulate ADRA2A/14-3-3γ temporal signal intensity differently, suggesting that certain drugs can differently modulate GPCR/14-3-3 complex trafficking and stability, a new approach for GPCR drug development.

## 2 Results

### 2.1 GPCRs can recruit different 14-3-3 isoforms in response to agonist treatment

There are seven 14-3-3 isoforms in humans. All 14-3-3 isoforms show significant structural homology (Fu et al., 2000). However, the different isoforms of 14-3-3 proteins may exert distinctive cellular functions, owing to their distinctive subcellular localization and expression profiles in different cell types (Smith et al., 2011). Previously, we investigated the 14-3-3ε isoform in GPCR/14-3-3 interaction assays (Yuan et al., 2019) using LinkLight assay technology (Eishingdrelo et al., 2011).

**Fig. 1:**
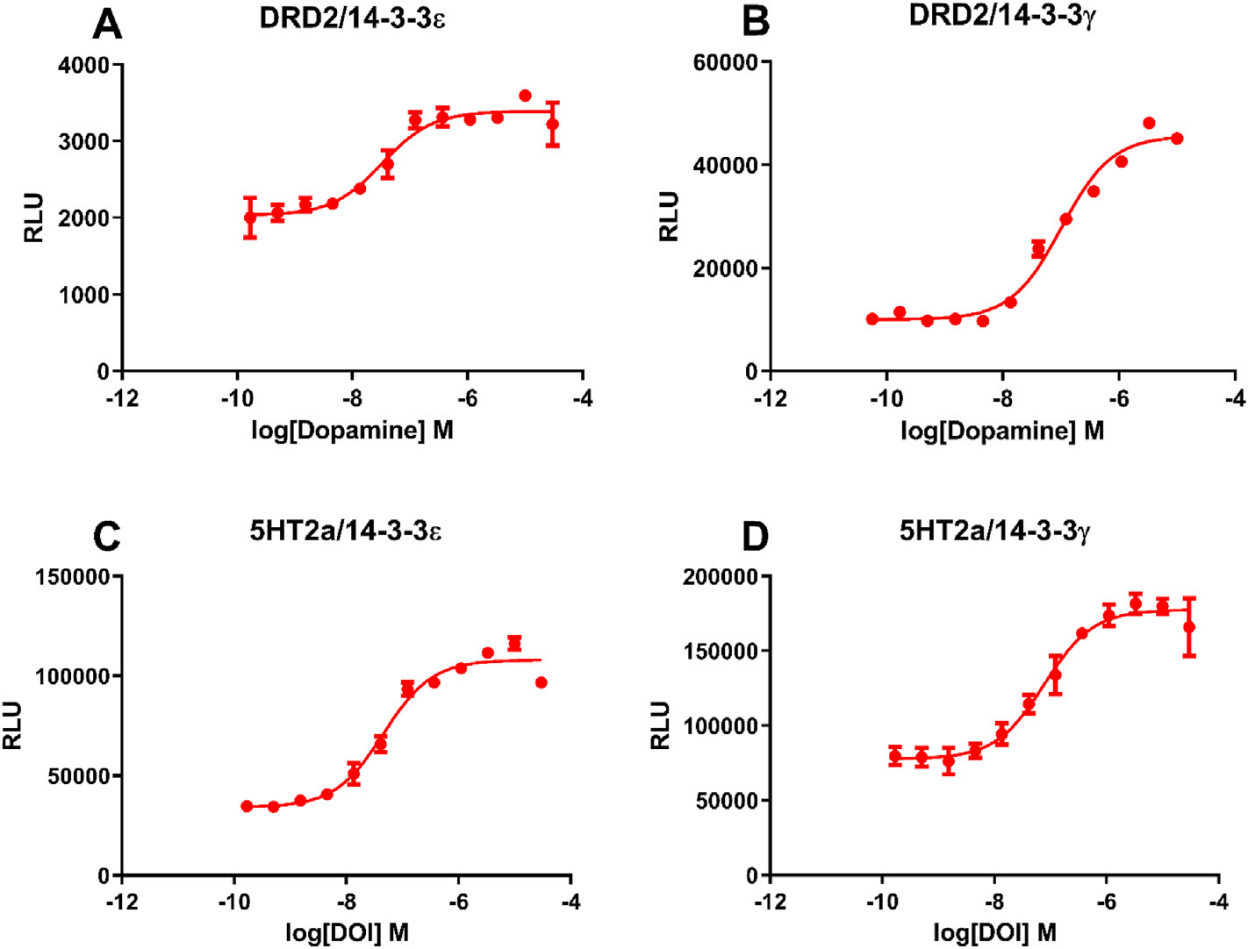
Agonist-induced GPCR interaction signals with different 14-3-3 isoforms. Concentration-response curves of GPCR/14-3-3 interaction signals for dopamine D2 receptor (DRD2), and serotonin (5-HT) receptor 2A (5HT2A) with 14-3-3ε (Fig. 1A and C) and 14-3-3γ (Fig. 1B and D) isoforms.

To see if a GPCR interacts with different 14-3-3 isoforms in the same or different manner, we selected the 14-3-3γ isoform for comparison to 14-3-3ε. The 14-3-3γ isoform was reported to participate in neural processes such as receptor trafficking and ion channel regulation (Cho and Park, 2020). We developed dopamine receptor 2 (DRD2) and 5-hydroxytryptamine receptor 2A (5HT2A) assays with both 14-3-3ε and 14-3-3γ isoforms. We first established stable 14-3-3ε-pLuc (permuted luciferase) and 14-3-3γ-pLuc reporter HEK293 ​cell lines, and then stably expressed DRD2-TEV (tobacco etch virus protease) or 5HT2A-TEV in the reporter cells. We found dopamine promoted both DRD2/14-3-3ε and DRD2/14-3-3γ signals (Fig. 1A and B) and similarly, serotonin (5-hydroxytryptamine) promoted both 5HT2A/14-3-3ε and 5HT2A/14-3-3γ signals (Fig. 1C and D). In both cases, an agonist can promote a GPCR interaction signals with different 14-3-3 isoforms in a concentration-dependent manner. These results indicate that a GPCR can recruit different 14-3-3 isoforms in response to agonist treatment.

### 2.2 GPCRs can differentially recruit one isoform and dissociate another isoform in response to agonist treatment

**Fig. 2:**
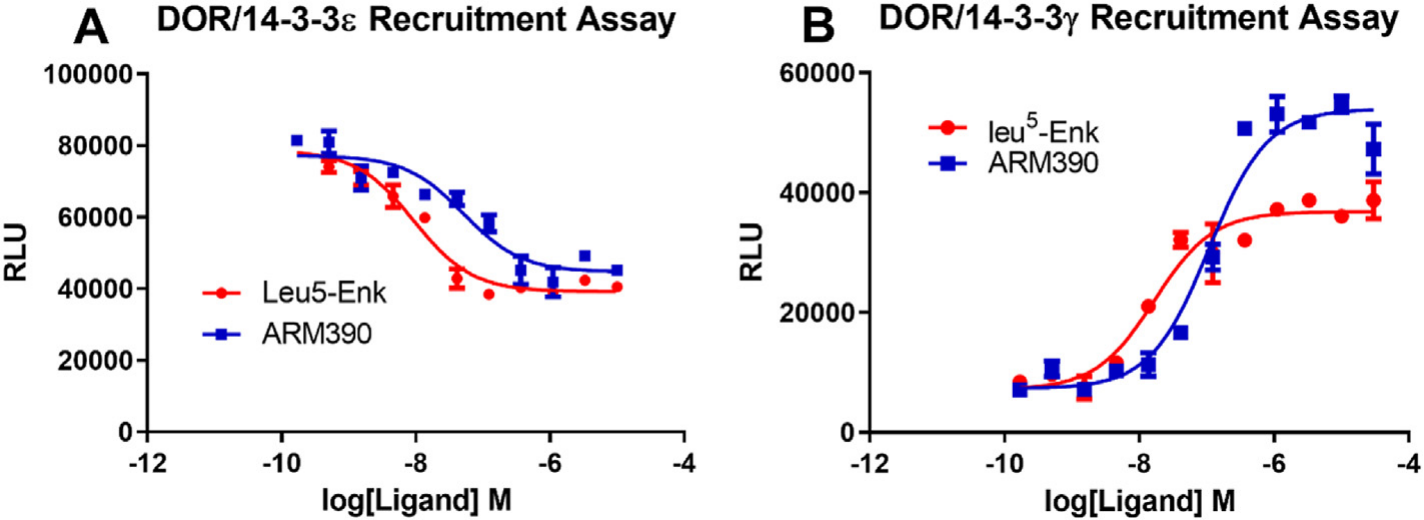
**Agonists can promote a GPCR interaction with one 14-3-3 isoform and decrease association with another 14-3-3 isoform.** Delta-opioid receptor (DOR) peptide agonist Leu^5^-enkephalin (Leu^5^-Enk) and small-molecule agonist ARM1000390 (ARM390) decrease DOR/14-3-3ε signals (Fig. 2A) but increase DOR/14-3-3γ signals (Fig. 2B) in a concentration-dependent manner.

Using the DOR/14-3-3ε LinkLight assay we previously developed (Yuan et al., 2019), we showed that opioid agonists Leu^5^-Enk and ARM390 diminished DOR/14-3-3ε interaction signals in a concentration-dependent manner (Fig. 2A) after 2 ​h of ligand treatment. The data suggest that DOR/14-3-3ε could form a complex in the absence of an agonist and that agonist-diminished signals would indicate either the decreased amount of DOR/14-3-3ε complex or lost DOR/14-3-3ε interaction. It is possible that the DOR/14-3-3ε complex is triggered by agonists to enter a degradation process, resulting in diminished signals. It is noteworthy that the agonist-mediated DOR/14-3-3ε signal change showed a concentration-dependent *decrease*, opposite of the agonist-induced *increase* in the aforementioned DRD2/14-3-3ε and 5HT2A/14-3-3ε signals. To examine whether the 14-3-3γ isoform behaves differently from the 14-3-3ε isoform when interacting with DOR, we utilized the DOR/14-3-3γ LinkLight assay. We generated stable DOR/14-3-3γ cells and investigated DOR/14-3-3γ interaction signals in response to agonist treatment. In contrast to the decreased interaction signals with DOR1/14-3-3ε, opioid agonists Leu^5^-Enk and ARM390 *increased* DOR/14-3-3γ interaction signals (Fig. 2B) after 2 ​h of ligand incubation. This is the first example to our knowledge that different 14-3-3 isoforms differentially interact with a GPCR. GPCRs have different abilities to recruit or dissociate 14-3-3 isoforms. The results suggest that different GPCR/14-3-3 isoform complexes (DOR/14-3-3ε and DOR/14-3-3γ) may play different roles in cellular signaling, and different 14-3-3 isoforms may serve distinctive functions.

### 2.3 GPCRs temporally engage with 14-3-3 proteins

**Fig. 3:**
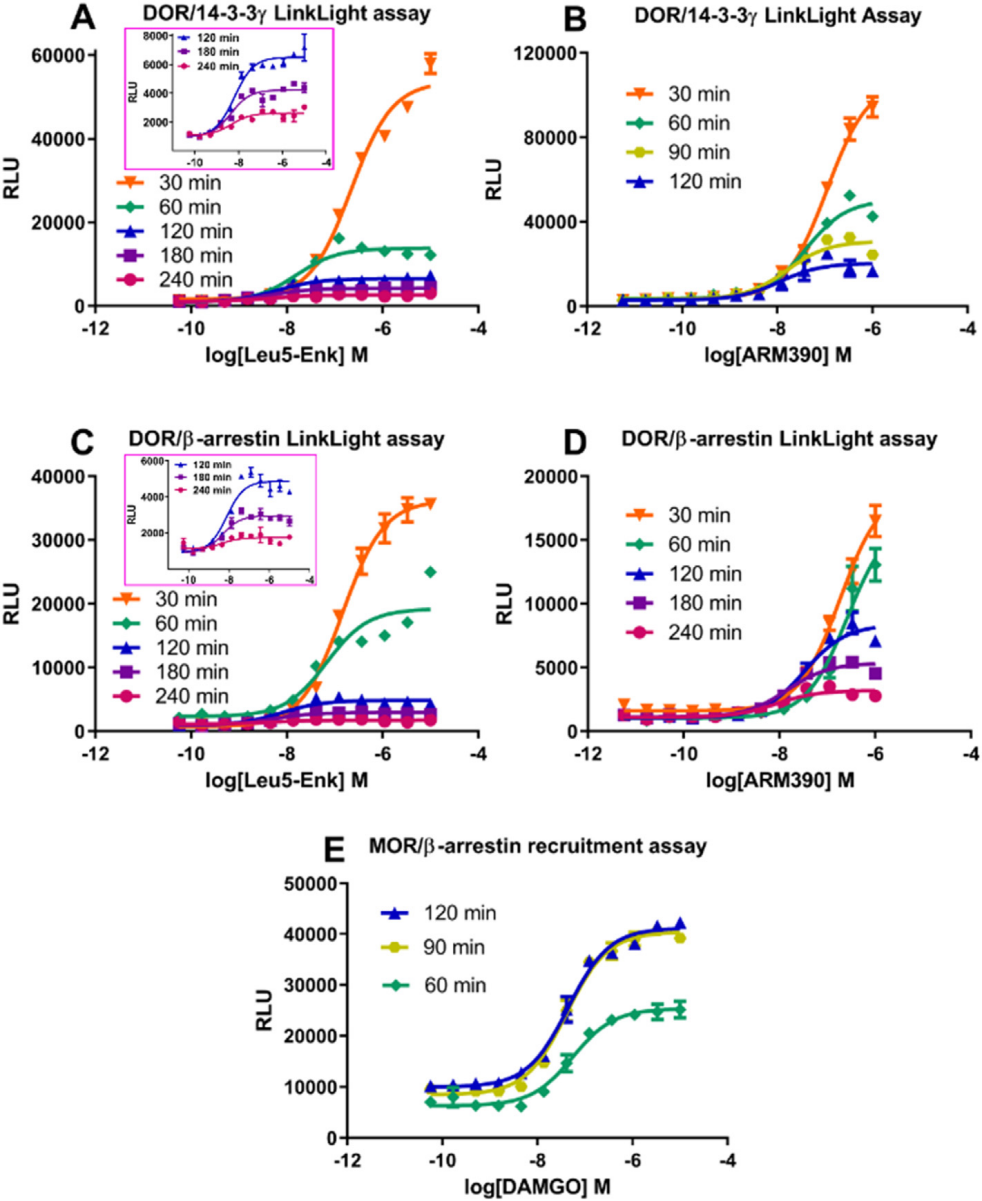
**Comparison of rapidly decreasing agonist-induced DOR/14-3-3γ and DOR/β-arrestin-2 signal intensities for different time points.** The timeframes of rapidly decreasing DOR/14-3-3γ signal intensities (concentration-response curves) by Leu^5^-Enk (Fig. 3A) and ARM390 (Fig. 3B). Fig. 3C and D shows the timeframes of rapidly decreasing DOR/β-arrestin-2 signal intensities (concentration-response curves) by Leu^5^-Enk (Fig. 3C) and ARM390 (Fig. 3D), respectively. Insets in Fig. 3A and C removed 30 and 60 ​min data points to show the details of later time point data. Fig. 3E shows MOR agonist DAMGO-induced mu-opioid receptor MOR/β-arrestin-2 signal intensity increased over time.

We investigated the time course of DOR/14-3-3γ interaction signals in response to agonist treatment. Unexpectedly, instead of signal intensity gradually increasing over time as we observed for many agonist-induced GPCR/14-3-3 interactions, the agonist-induced DOR/14-3-3γ interaction signal intensity rapidly diminished over time. This is the case for both peptide and small molecule agonists Leu^5^-Enk and ARM390 (Fig. 3A and B; the figure inset in Fig. 3A removed 30 and 60 ​min data points to show details of later time points). The results prompted us to investigate the time course of DOR/β-arrestin-2 interaction signals. Likewise, DOR/β-arrestin interaction signal intensity also rapidly diminished over time in response to agonist treatment (Fig. 3C and D; the figure inset in Fig. 3C removed 30 and 60 ​min data points to show details of later time points). The characteristic of time frame-related signal intensity diminishing was similar for both DOR/14-3-3γ and DOR/β-arrestin interactions, suggesting that DOR recruits β-arrestin-2 and 14-3-3γ within the same time frame, and that the amounts of DOR/14-3-3y and DOR/β-arrestin-2 complexes were quickly reduced. Previously, we observed agonist-diminished MOR/14-3-3ε signals (Yuan et al., 2019). We performed transient expression of MOR-TEV in 14-3-3γ-pLuc reporter cells but did not observe agonist-altered signals (no change in dose-response relationship was observed; data not shown). Instead, we observed that MOR/β-arrestin interaction signals were enhanced over time, reaching a plateau after 90 ​min of agonist incubation (Fig. 3E). DOR/14-3-3ε basal signals did not diminish in response to agonist treatment for 3 ​h (no change in dose-response relationship was observed; data not shown). These results suggest that DOR and MOR have different trafficking patterns (Wang and Pickel, 2001; von Zastrow et al., 2003). The rapidly diminishing DOR/14-3-3γ and DOR/β-arrestin interaction signals could mean that DOR/14-3-3γ and DOR/β-arrestin complexes are targeted to lysosomes for degradation. GPCR/β-arrestin recruitment signals have been regarded as an endocytosis marker. The persistent MOR/β-arrestin complex signal intensity vs, quickly diminished DOR/β-arrestin-2 and DOR/14-3-3γ signal intensity indicate that MOR and DOR have different trafficking patterns, as previously reported (Wang and Pickel, 2001; von Zastrow et al., 2003) that preferential membrane plasma MOR localization and cytoplasmic localization of DOR.

### 2.4 Different ligands can temporally modulate GPCR/14-3-3 interaction

**Fig. 4:**
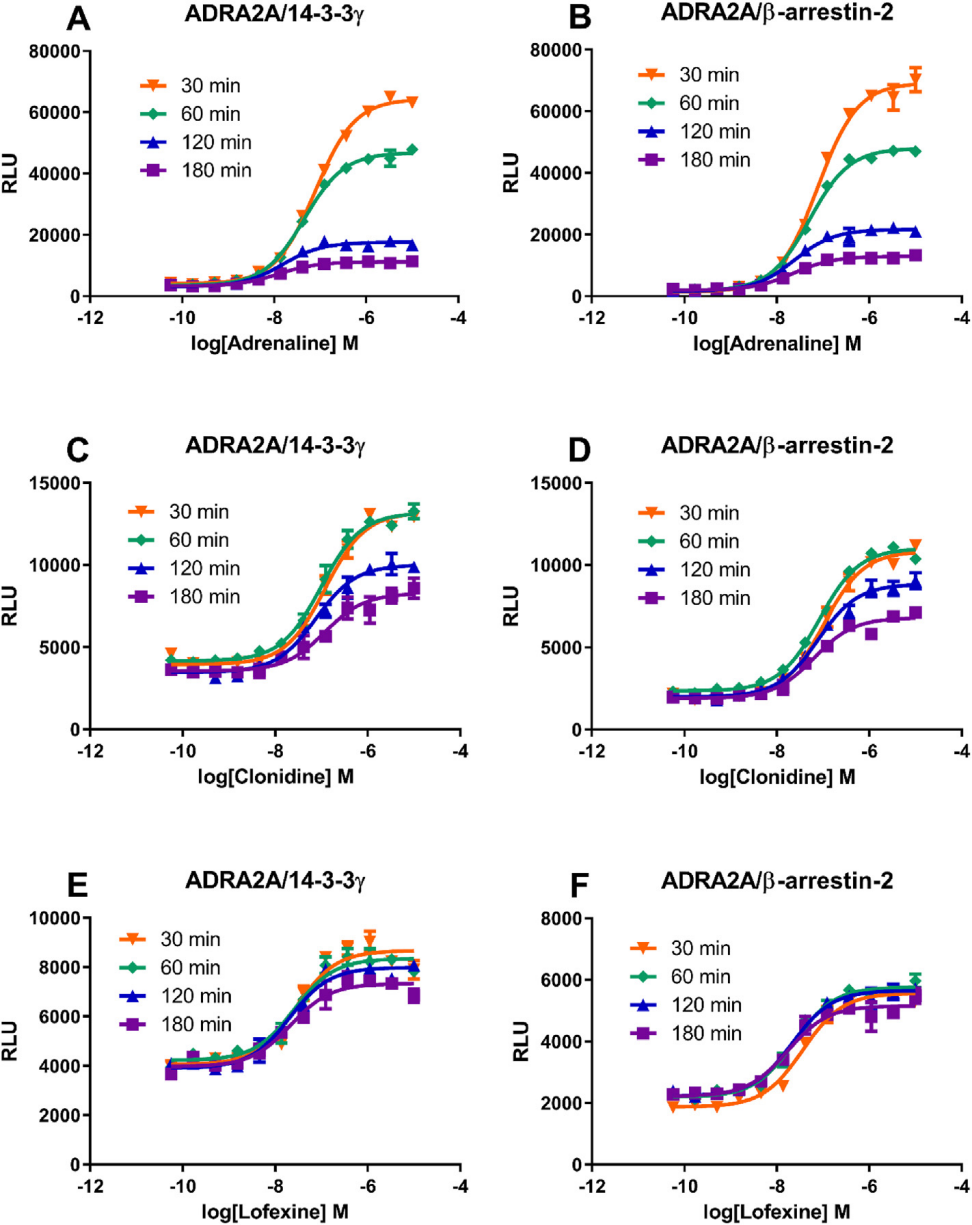
**Temporal regulation of alpha-adrenergic receptor 2A ADRA2A/14-3-3γ and ADRA2A/β-arrestin-2 signal intensities by different ligands.**Fig. 4A and B, different time points of ADRA2A/14-3-3γ and ADRA2A/β-arrestin-2 signal intensities induced by adrenaline. Fig. 4C and D, different time points of ADRA2A/14-3-3γ and ADRA2A/β-arrestin-2 signal intensities induced by clonidine. Fig. 4E and F, different time points of ADRA2A/14-3-3γ and ADRA2A/β-arrestin-2 signal intensities induced by lofexidine.

A compound that can alter a GPCR/14-3-3 signal intensity temporally may alter the GPCR trafficking pattern and affect its signaling. We were interested to see if a drug can alter a GPCR/14-3-3 signal intensity temporally. Since the adrenergic receptor alpha 2A (ADRA2A) has a large number of readily available agonist drugs, we developed an ADRA2A/14-3-3γ recruitment assay. The ADRA2A does not internalize after agonist treatment (Daunt et al., 1997). Similar to the observation that agonist-induced DOR/14-3-3γ signal intensity diminished rapidly, the ADRA2A/14-3-3γ interaction signal intensity also quickly diminished over time (Fig. 4A). The same phenomenon was also observed in the ADRA2A/β-arrestin-2 LinkLight assay (Fig. 4B). The quickly diminished signal intensity suggests quickly disappearing ADRA2A/14-3-3γ and ADRA2A/β-arrestin-2 complexes during endocytosis, likely going through a degradation process in lysosomes. We tested different ADRA2A agonists in both ADRA2A/14-3-3γ and ADRA2A/β-arrestin-2 assays, and looked for an agonist that could maintain the signal intensity. Adrenaline was used as a standard reference agonist (Fig. 4A and B). Clonidine is in a class of medications acting on ADRA2A as an agonist, and it has been approved for the treatment of high blood pressure and attention deficit hyperactivity disorder. Lofexidine is an ADRA2A agonist that was recently approved by the FDA for the symptomatic treatment of acute opioid withdrawal syndrome. Our data showed that adrenaline-induced ADRA2A/14-3-3γ and ADRA2A/β-arrestin-2 signal intensity quickly decreased (Fig. 4A and B); the same is true for clonidine-induced ADRA2A/14-3-3γ and ADRA2A/β-arrestin-2 signal intensity (Fig. 4C and D). However, Lofexidine-induced ADRA2A/14-3-3γ and ADRA2A/β-arrestin-2 signal intensity was long-lasting (Fig. 4E and F), indicating that lofexidine can alter ADRA2A/14-3-3γ and ADRA2A/β-arrestin-2 complex trafficking or stability. It should be pointed out that, although the signal strength rapidly decreased, the EC_50_ values in both ADRA2A/14-3-3γ and ADRA2A/β-arrestin-2 assays were little changed. These results suggest that different ligands can temporally alter ADRA2A signaling activity in distinct ways.

## 3 Discussion

14-3-3 proteins are highly expressed in the brain and broadly distributed throughout the subcellular organelles including the plasma membrane, endosomes, endoplasmic reticulum, Golgi apparatus, nucleus, centrosomes, microtubules, mitochondria, and actin fibers (Abdrabou et al., 2020). 14-3-3 proteins participate in a vast array of physiological processes such as neurotransmission, neurite growth, neuroplasticity, cell motility, cell cycle, cell proliferation, and apoptosis (Darling et al., 2005). 14-3-3 proteins interact with a remarkably large number of diverse proteins including receptors, channels, kinases, metabolic enzymes, transcriptional factors, signal adaptors, and filaments, serving as transporters, adapters, activators, and repressors (Fu et al., 2000). Given the broad roles of 14-3-3 proteins, non-selective 14-3-3 compounds may have limited therapeutic potential. In contrast, modulating a specific target/14-3-3 pathway may offer the opportunity to fine-tune a subset of signaling processes. Thus, targeting specific GPCR/14-3-3 pathway could identify compounds with physiological functions different from traditional GPCR drugs signaling through G-proteins or β-arrestin.

All 14-3-3 isoforms show structural similarities (Ferl et al., 2002), suggesting that 14-3-3 isoforms could have functional overlap and the ability of one isoform to compensate for the loss of another isoform. However, the tissue preferential expression, differential subcellular localization, and isoform-specific motifs of 14-3-3 suggest that a substantial extent of functional specialization does exist (Freeman and Morrison, 2011). Therefore, an important question is, is a receptor able to interact with different 14-3-3 isoforms within the same cell? Based on our results, the answer is yes. A GPCR can interact with different 14-3-3 isoforms in response to agonist treatment in the same cells, as we observed agonist-induced DRD2/14-3-3ε and DRD2/14-3-3γ signals, as well as agonist-induced 5HT2A/14-3-3ε and 5HT2A/14-3-3γ signals. It is noteworthy that the concentration-response curve shapes of DRD2/14-3-3ε and DRD2/14-3-3γ are different: the DRD2/14-3-3ε curve is shallower, and the curve of DRD2/14-3-3γ is steeper. The shallow concentration-response curve of DRD2/14-3-3ε indicates that a large change in agonist dosing is needed to cause an appreciable increase in the biological response, whereas the steeper concentration-response curve of DRD2/14-3-3γ indicates that a relatively mild change in agonist dosing is sufficient to cause an increase in the biological response. Although a GPCR can recruit different 14-3-3 isoforms, it appears that a GPCR has a different affinity to 14-3-3 isoforms.

Previously, we observed that agonists can diminish GPCR/14-3-3ε signals (Yuan et al., 2019), suggesting that agonists can also down-regulate GPCR/14-3-3 signals, not just promote GPCR/14-3-3 complex formation. We wondered if a receptor could interact with 14-3-3 isoforms differentially? We observed that DOR/14-3-3ε signals were decreased by agonist treatment. Interestingly, unlike agonist-decreased DOR/14-3-3ε signals, DOR/14-3-3γ signals were ***increased*** by both peptidic and small molecular agonists. This is the first report that a GPCR can differentially engage with different 14-3-3 isoforms in response to agonist treatment: promoting recruitment of a 14-3-3 isoform and dissociating from another pre-assembled 14-3-3 isoform/GPCR complex. The phenomenon could be explained by the different subcellular locations of DOR/14-3-3ε and DOR/14-3-3γ complexes. It is likely that ligands activate membrane DOR, and activated DOR recruit 14-3-3γ to undergo endocytosis, while the DOR/14-3-3ε complex could be pre-assembled in the cytoplasmic or endoplasmic reticulum locations without the presence of an agonist. Activation of DOR could affect the stability or mobility of the DOR/14-3-3ε complex in the cytoplasm or endoplasmic reticulum, or Golgi, resulting in diminished DOR/14-3-3ε basal interaction signals over time. To confirm agonist-promoted DOR/14-3-3γ signals that occur in the endocytosis, we performed the DOR/β-arrestin-2 recruitment assay, since GPCR recruitment of β-arrestins is a marker for GPCR endocytosis. Both peptidic and small molecular agonists produced agonist-induced DOR/14-3-3γ and DOR/β-arrestin-2 signals over the same timeframe. These results imply that DOR trafficking and subcellular localization can be differentially regulated by modulating its interaction with different 14-3-3 isoforms. The fact that a GPCR can interact with multiple 14-3-3 isoforms represents one more level of regulation in GPCR signaling. Different 14-3-3 isoform expression levels and cell types that express a specific dominant 14-3-3 isoform may explain some of the conflicting reports of DOR cellular localizations (Gendron et al., 2006; He et al., 2021; Gillis and Christie, 2021).

Also reported here for the first time is the observation that agonist-induced GPCR/14-3-3 signal intensity can quickly decrease. The DOR/14-3-3γ signal intensities rapidly decreased over 30, 60, 120, and 180-min time points. This is in contrast to our previous observation that agonist-induced GPCR/adaptor signal intensity increased over time. The quick decrease of DOR/14-3-3γ signal intensity may reflect the decreased amount of the DOR/14-3-3γ complex. It could be due to the quick degradation of ligand-activated DOR in lysosomes. If DOR has a quick degradation process, we should observe the same phenomenon in the DOR/β-arrestin-2 assay. Indeed, the agonist-induced DOR/β-arrestin-2 signal intensity also rapidly decreased, the same as the rapidly decreased DOR/14-3-3γ signal intensity. Previous studies showed that agonists promote DOR phosphorylation, leading to its degradation (Trapaidze et al., 2000; Gaudriault et al., 1997; Hislop et al., 2009). The quick decrease of DOR/signal adaptor signal intensity supports the observation that ligand-activated DOR undergoes a quick degradation process. Previous studies also suggested that the trafficking patterns of DOR and MOR are different (Wang and Pickel, 2001; von Zastrow et al., 2003). This prompted us to investigate MOR/signal adaptor signal intensity over time. Previously, we showed that MOR/14-3-3ε signals decreased in response to agonist treatment over time (Yuan et al., 2019). Here we observed that the MOR/β-arrestin-2 signal intensity was increased over time, reaching a plateau around 90–120 ​min after agonist incubation. Our results suggest that DOR/14-3-3γ and DOR/β-arrestin complexes are targeted to lysosomes for degradation, while MOR/β-arrestin complexes with persistent signals participate in recycling/trafficking and other cellular processes.

Can we temporally regulate GPCR/signal adaptor signaling? Compounds that can alter quickly-decreased agonist-induced GPCR/adaptor signal intensity may alter GPCR trafficking patterns and affect GPCR signaling. Such compounds could offer unique therapeutic value. We used ADRA2A as an example to find existing drugs that can differentially modulate ADRA2A/adaptor signal intensity temporally. Similar to the situation where agonist-induced DOR/14-3-3γ signal intensity diminished rapidly, the ADRA2A/14-3-3γ interaction signal intensity also quickly diminished. The rapid signal intensity decrease was also observed in the ADRA2A/β-arrestin-2 LinkLight assay. The quickly diminished signal intensity suggests quickly disappearing ADRA2A/14-3-3γ and ADRA2A/β-arrestin-2 complexes, likely undergoing a degradation process. We looked for a drug that could maintain the signal intensity over time. We used adrenaline as a standard reference agonist. Clonidine is in a class of medications acting on ADRA2A as an agonist and has been approved for treating high blood pressure and attention deficit hyperactivity disorder. Lofexidine is another ADRA2A agonist and was recently approved by the FDA for the symptomatic treatment of acute opioid withdrawal syndrome. Our data showed that adrenaline-induced ADRA2A/14-3-3γ and ADRA2A/β-arrestin-2 signal intensity quickly decreased; while lofexidine-induced ADRA2A/14-3-3γ and ADRA2A/β-arrestin-2 signal intensity were long-lasting, indicating that lofexidine can alter ADRA2A/14-3-3γ and ADRA2A/β-arrestin-2 complex trafficking or stability, linking ADRA2A trafficking with antidepressant pharmacology (Cottingham et al., 2015). Our results indicate that compounds can regulate GPCR/adaptor temporally. Such compounds may have different therapeutic utilities. In addition, our results suggest a new approach for drug development in that one can look for compounds temporally altering GPCR trafficking and stability, and such compounds could possess a new pharmacologic property.

## 4 Materials and methods

### 4.1 Compounds and chemicals

Compounds and chemicals were purchased from Sigma-Aldrich (St. Louis, MO, USA), ApexBio (Houston, Texas, USA), AOBIOUS (Gloucester, MA, USA), and Tocris Biosciences (Bristol, UK).

### 4.2 LinkLight assay

The cell-based protein-protein interaction LinkLight assay consists of two components (Supplemental Fig. 1). A pLuc (permuted luciferase) is created by breaking luciferase into two fragments, rearranging the fragment order in that the N-terminal fragment is moved to the C-terminus and the C-terminal fragment is moved to the N-terminus, and reconnecting them by a TEV (tobacco etch virus protease) protease cleavage sequence. This permuted luciferase is linked to the C-terminus of a 14-3-3 protein. A TEV protease is linked to the C-terminus of a GPCR. Upon interaction between GPCR and 14-3-3, inactive pLuc is cleaved, the cleaved luciferase fragments are spontaneously refolded, driven by fragment self-complementation affinity, and active luciferase is reconstituted. The technology does not require strict spatial orientation requirements for both interacting partners and complemental fragments. It also overcomes the complemental fragment high-affinity issue that can drive irreversible complementation that causes false interaction or high background signals.

### 4.3 Cell lines and cell culture

HEK293 ​cells were routinely maintained and passaged in standard DMEM with 10% FBS and Pen/Strep (Gibco Catalog # 15070). Cells were cultured in a 37 ​°C incubator with 5% CO_2_. The cell culture medium was replaced every three to four days, and cells were passaged at 90% confluence. Stable GPCR/14-3-3 and GPCR/β-arrestin LinkLight cells are maintained with HEK293 culture media with 400 ​μg/ml G418 and 100 ​μg/ml Hygromycin B.

### 4.4 Plasmid construction and generation of stable cell lines

Full-length cDNAs of human GPCRs without a stop codon were subcloned in frame with the TEV protease vector (cat.#: V-002, BioInvenu) as previously described (Eishingdrelo et al., 2011). The β-arrestin-2-permuted luciferase expression report cell line (cat. #: RL-009) was also previously described (Eishingdrelo et al., 2011). 14-3-3ϵ and 14-3-3γ full-length cDNAs without a stop codon were used to replace β-arrestin-2 in the β-arr-2-pLuc for the construction of the 14-3-3ϵ-pLuc and 14-3-3γ-pLuc expression plasmids. Transfections of HEK293 ​cells were performed with PEI transfection reagent (catalog # 23966-1, Polysciences). Monoclonal 14-3-3ϵ-pLuc and 14-3-3γ-pLuc report cell lines (cat.# RL-07 and RL-33, BioInvenu) were selected using 400 ​μg/ml G418 (Invitrogen/Life Technologies, catalog # 10131-027). Generation of GPCR/14-3-3 monoclonal cell lines was done by transfecting report cell lines with a GPCR-TEV expression plasmid with a Hygromycin selection marker. Multiple GPCR/14-3-3 or GPCR/β-arrestin-2 monoclonal cell lines were selected using 400 ​μg/ml G418 and 100 ​μg/ml Hygromycin B (Life Technologies, catalog # 10687-010), and evaluated for the best signal/background signal ratio in response to agonist treatment.

### 4.5 Luciferase assay

The cells expressing firefly luciferase were seeded into a 384-well white and sterile plate (Becton Dickinson, cat#: 356660) at 20,000 ​cells per well in 20 ​μl of the culture medium without antibiotics. Cells were cultured overnight. The next day, the cells were treated with GPCR ligands (5 μl/well) and incubated at room temperature for 120 ​min or for other times as indicated (time-course experiments). An equal volume of luciferase detection reagents Luci-Glo (cat. #: L0100, BioInvenu) was added to each well. The luminescence signals were recorded using a luminescence plate reader (EnSpire or EnVision). Optimum luminescent signals (relative light units) were observed between 2 and 15 ​min after adding the luciferase detection reagent.

### 4.6 Measurement of the effects of ligands on GPCR/14-3-3 signaling

The stably expressing GPCR/14-3-3 isoform and GPCR/β-arrestin-2 LinkLight cells were seeded into a 384-well white and sterile plate (Becton Dickinson, cat#: 356660) at 20,000 ​cells per well in 20 ​μl of the culture medium without antibiotics. Cells were cultured overnight. After overnight culture, 5 ​μl of a serial 1:3 dilution of GPCR ligand was added to each well in triplicate data points. After agonist incubation at room temperature (120 ​min–180 ​min, unless otherwise specified at designated time points), a luciferase detection reagent (20 μl/well) was added to the cells, and luminescence signals were recorded.

### 4.7 Data analysis

Concentration-response curves were analyzed using Prism software (GraphPad Software, Inc., San Diego, CA). All compounds were started with 10 ​μM, then a serial 1:3 fold dilutions. Assay quality was monitored by calculation of the Z′ factor (>0.6–1). Curves were fit by nonlinear regression using the sigmoidal dose-response equation in GraphPad Prism version 4.03 (GraphPad Software, Inc., San Diego, CA). All graphs (RLU vs. concentration) and data points shown are mean values ​± ​SEM obtained from at least two independent experiments performed in triplicate.

## Author contributions

L.Y. contributed to cell line generation and assay development experiments. X.Q. contributed to the assays. S.K. and L.Y. contributed to analyzing the results and to the manuscript preparation. H.E. contributed to the overall experimental design, assay development, and data analyses and took the leading role in writing the manuscript.

## Data and materials availability

The "target-open" LinkLight assay system in which researchers can generate assays for their target of interest as well as the aforementioned assays can be purchased from BioInvenu under a Material Transfer Agreement.

## Declaration of competing interest

The authors declare the following financial interests/personal relationships which may be considered as potential competing interests: Haifeng Eishingdrelo reports financial support was provided by National Institutes of Health. Haifeng Eishingdrelo reports a relationship with BioInvenu Corporation that includes: board membership, employment, equity or stocks, and funding grants. Haifeng Eishingdrelo has patent licensed to BioInvenu.
