# Supplementary material for: Ligands can differentially and temporally modulate GPCR interaction with 14-3-3 isoforms
Source: Curr Res Pharmacol Drug Discov. 2022 Aug 7;3:100123. doi: 10.1016/j.crphar.2022.100123 (PMC9389249; doi:10.1016/j.crphar.2022.100123)
Supplement: Multimedia component 1 [file mmc1.pdf]

Supplemental Figure: LinkLight assay technology

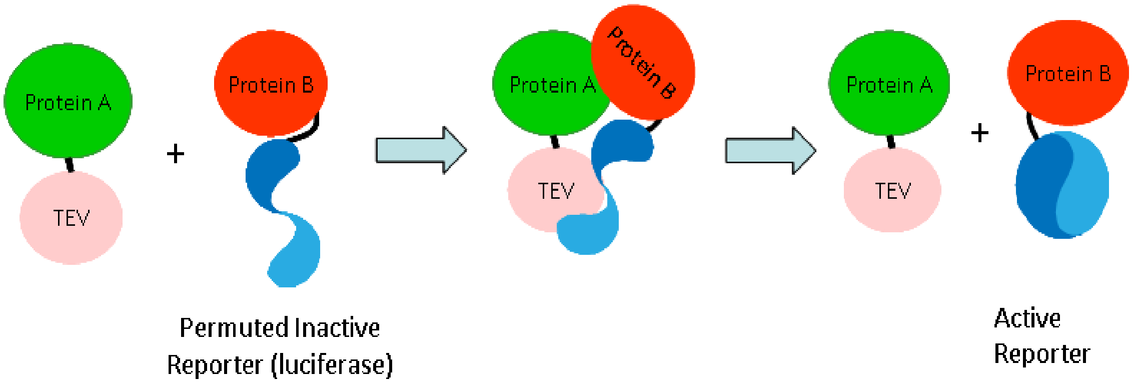

LinkLight assay consists of two components. A protein A is fused to a Tobacco Etch Virus (TEV) protease and a protein B is fused to an inactive permuted luciferase. The inactive permuted luciferase is created by breaking a luciferase coding sequence into two fragments, rearranging the fragment order in that the N-terminal fragment sequence is moved to the C-terminus and the C-terminal fragment sequence is moved to the N-terminus, and reconnecting them by a TEV protease cleavage sequence. The two components are expressed in live cells. Upon protein A and B interaction, inactive permuted protein is cleaved, the cleaved fragment spontaneously refold, driving by the high affinity of self-complementation force, and reconstitute an active reporter protein. The regenerated signal is specific for protein A and protein B interaction. The technology has advantages in that it does not require strictly spatiol and orientation aligment for both interaction partners and complenatation fragments. It also avoid high affinity force to drive irreversible two report fragment complementation which cause high background, low sensitivity, and false interaction signals.
